# Supplementary figures and images for: APRIL/BLyS deficient rats prevent donor specific antibody (DSA) production and cell proliferation in rodent kidney transplant model
Source: PLoS One. 2022 Oct 13;17(10):e0275564. doi: 10.1371/journal.pone.0275564 (PMC9562156; doi:10.1371/journal.pone.0275564)

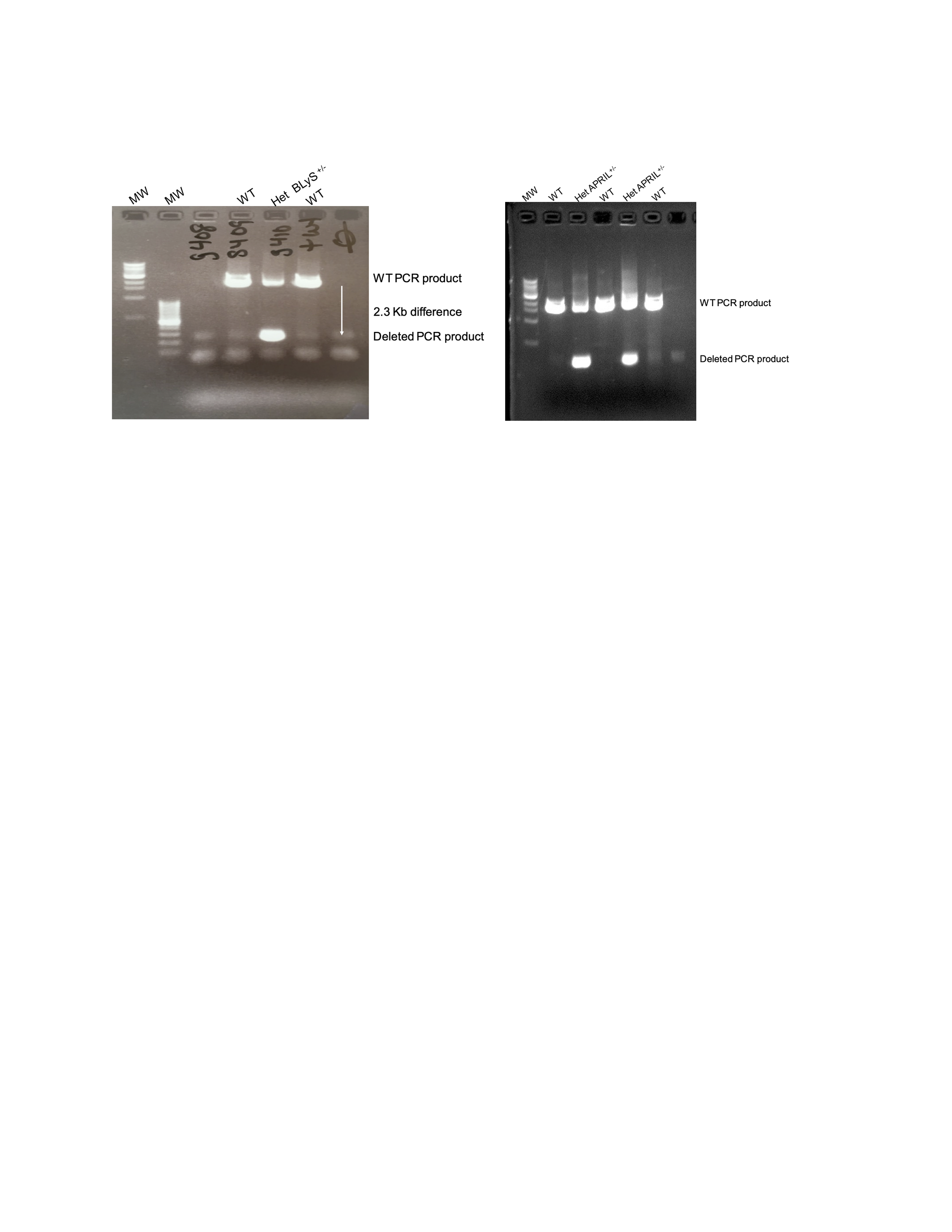

Supplement: S1 Raw images — (TIF) [file pone.0275564.s001.tif]

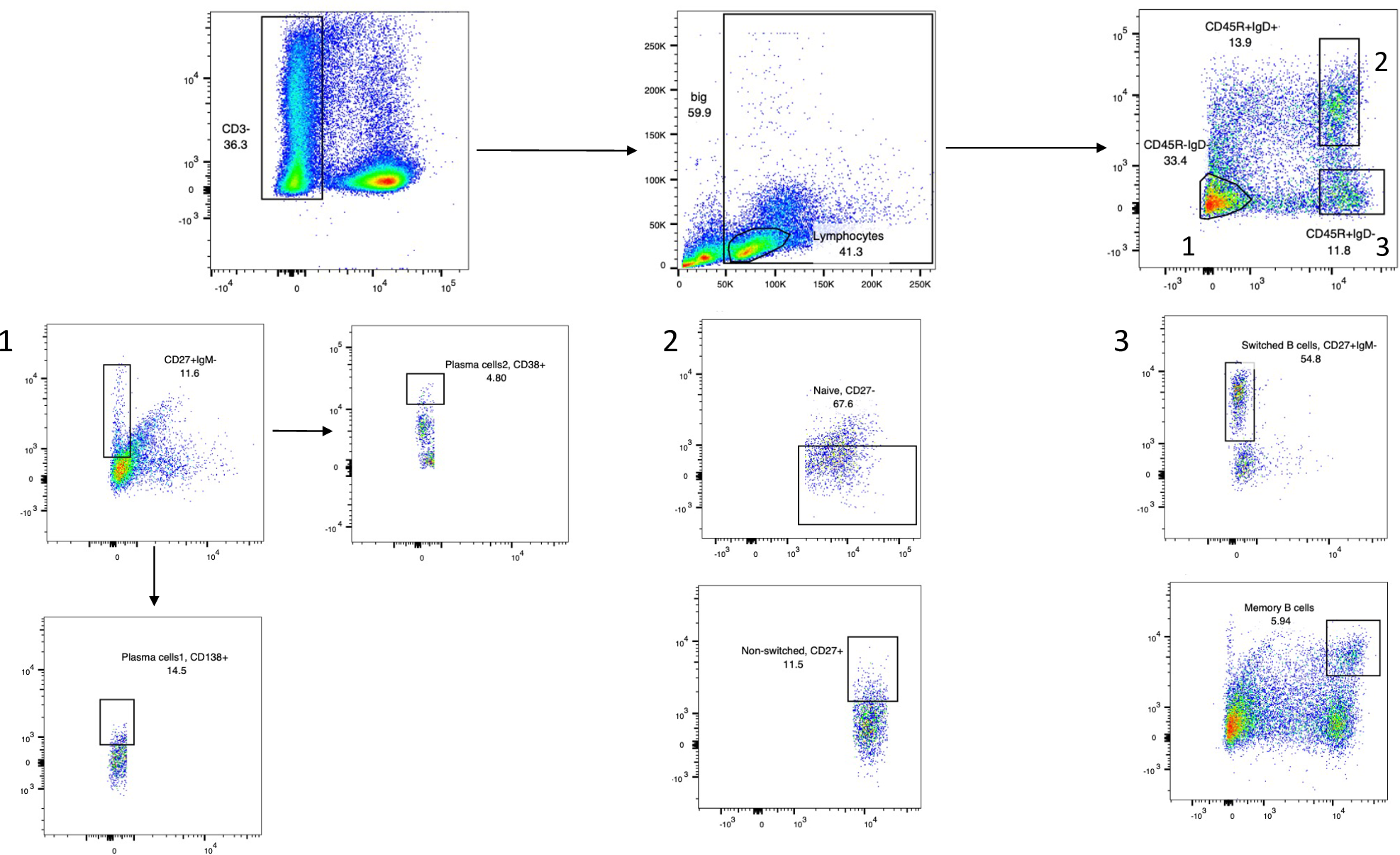

Supplement: S1 Fig — Top row: CD3- cells are selected from which lymphocytes are defined (forward versus side scatter). From this tight lymphocyte gate, cells are visualized as IgD versus CD45R. Cell populations that arise from here are defined as (1) CD45R-IgD-, (2) CD45R+IgD+, and (3) CD45R+IgD-. Gating in this image all comes from WT rodent. (1) From CD45R-IgD-, CD27+IgM- cells are gated. These cells are further identified as plasma cells if they are CD138+ or CD38+. Arrows indicate that cell populations originated from population gated in prior graph. (2) From CD45R+IgD+, naïve B lymphocytes are defined as CD27-IgD+ or non-switched B lymphocytes are defined separately from the CD45R+IgD+ as CD27+CD45R+. (3) From CD45R+IgD-, switched B lymphocytes are defined as CD27+IgM-. From CD45R+IgD-, memory B lymphocytes are defined as CD27+CD45R+. (TIF) [file pone.0275564.s002.tif]

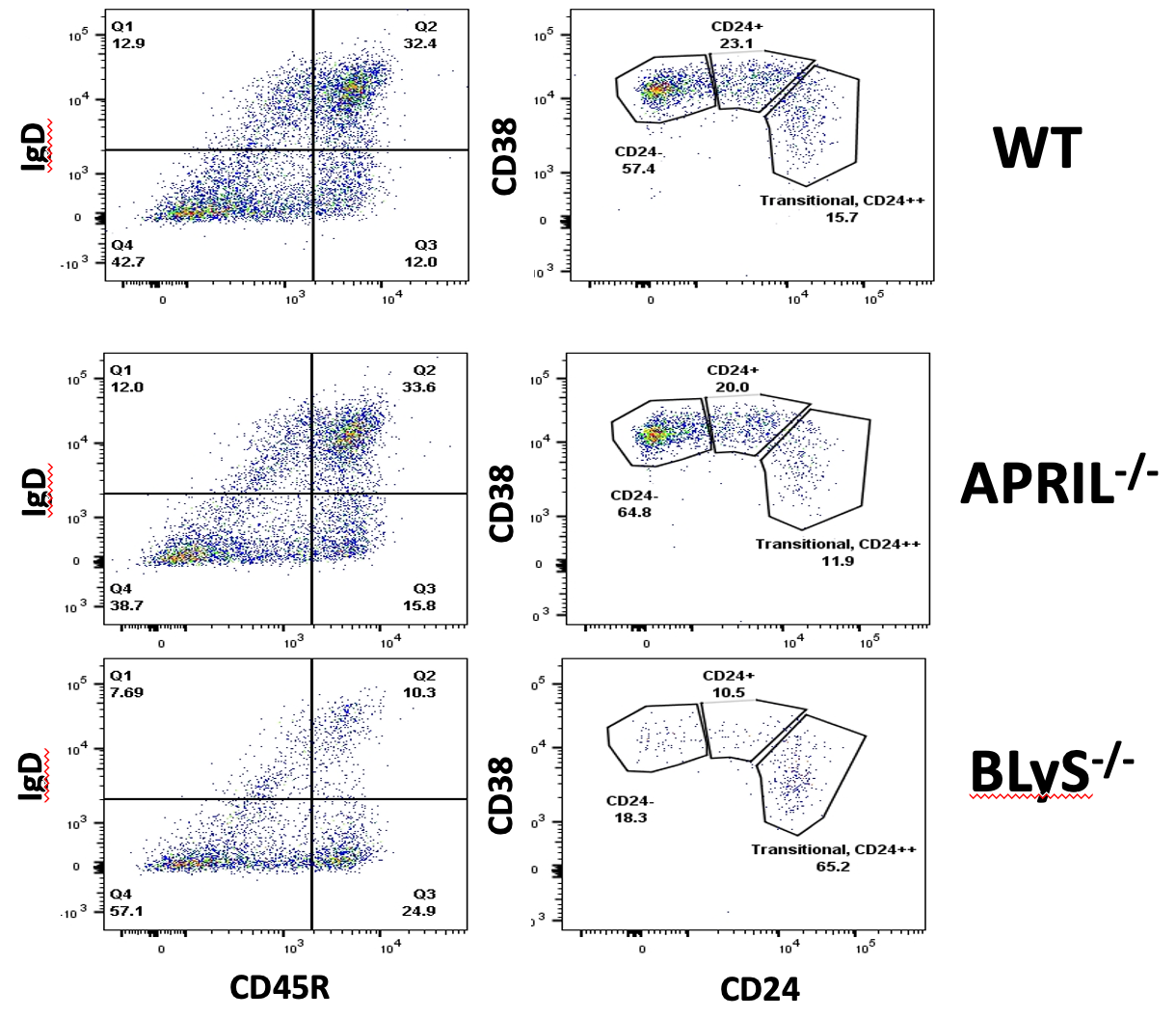

Supplement: S2 Fig — Gating strategy of TZ B lymphocytes demonstrated. After selecting CD3- lymphocytes from previous gates. Cells are visualized as IgD versus CD45R. IgD+CD45R+ gate (Q2) is selected and visualized as CD38 versus CD24. TZ B lymphocytes are defined as CD38+CD24++. (TIF) [file pone.0275564.s003.tif]
